# Supplementary material for: Upregulation of DR5 and Downregulation of Survivin by IITZ-01, Lysosomotropic Autophagy Inhibitor, Potentiates TRAIL-Mediated Apoptosis in Renal Cancer Cells via Ubiquitin-Proteasome Pathway
Source: Cancers (Basel). 2020 Aug 21;12(9):2363. doi: 10.3390/cancers12092363 (PMC7564912; doi:10.3390/cancers12092363)
Supplement: Supplementary file 1 [file cancers-12-02363-s001.pdf]

Supplementary Materials

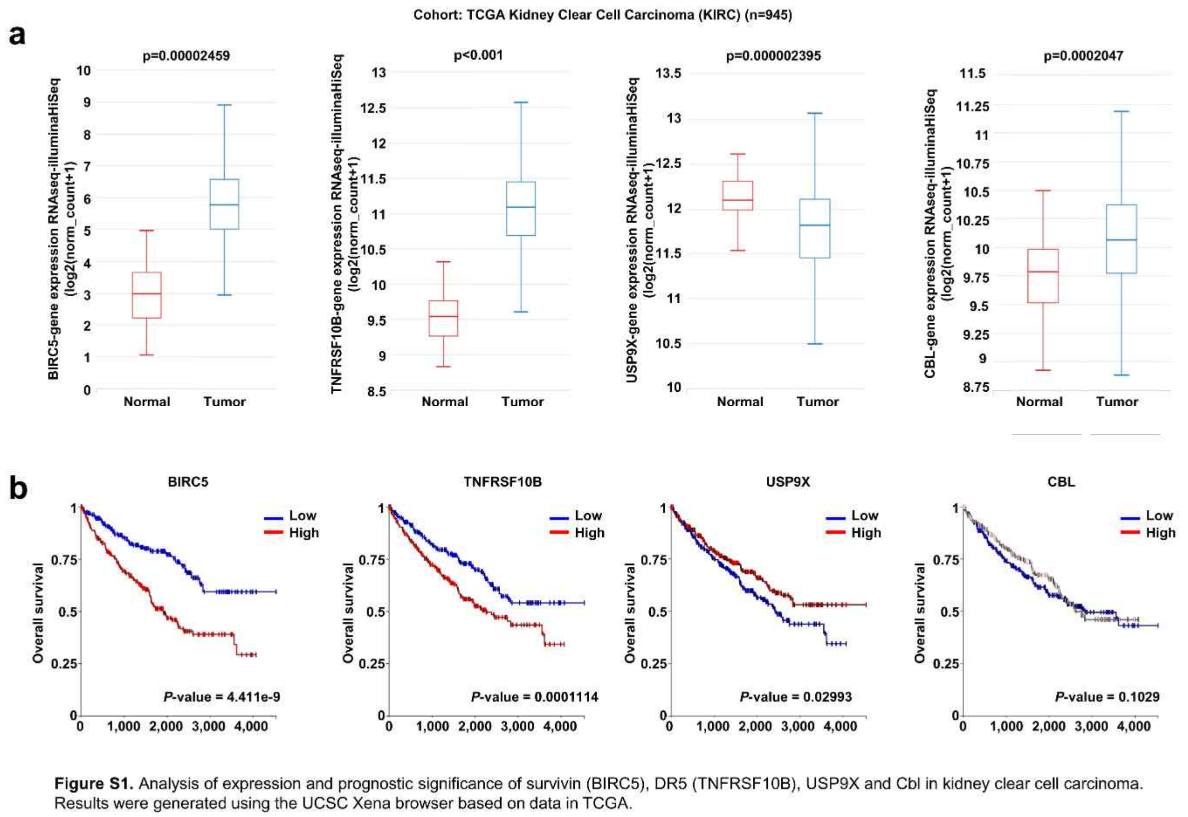

Figure S1. Analysis of expression and prognostic significance of survivin (BIRC5), DR5 (TNFRSF10B), USP9X and Cbl in renal clear cell carcinoma. Results were generated using the UCSC Xena browser based on data in TCGA.

Figure 1a

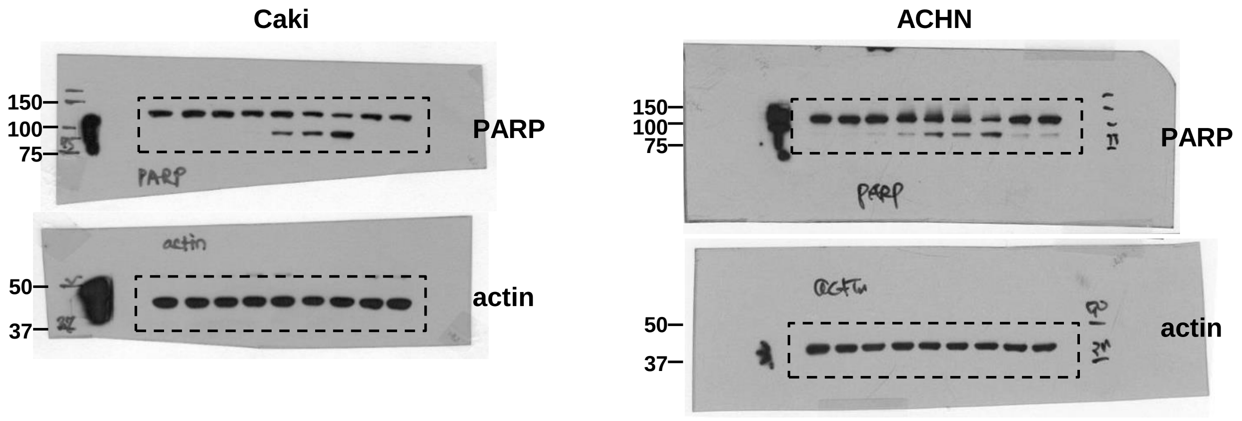

Figure 1e

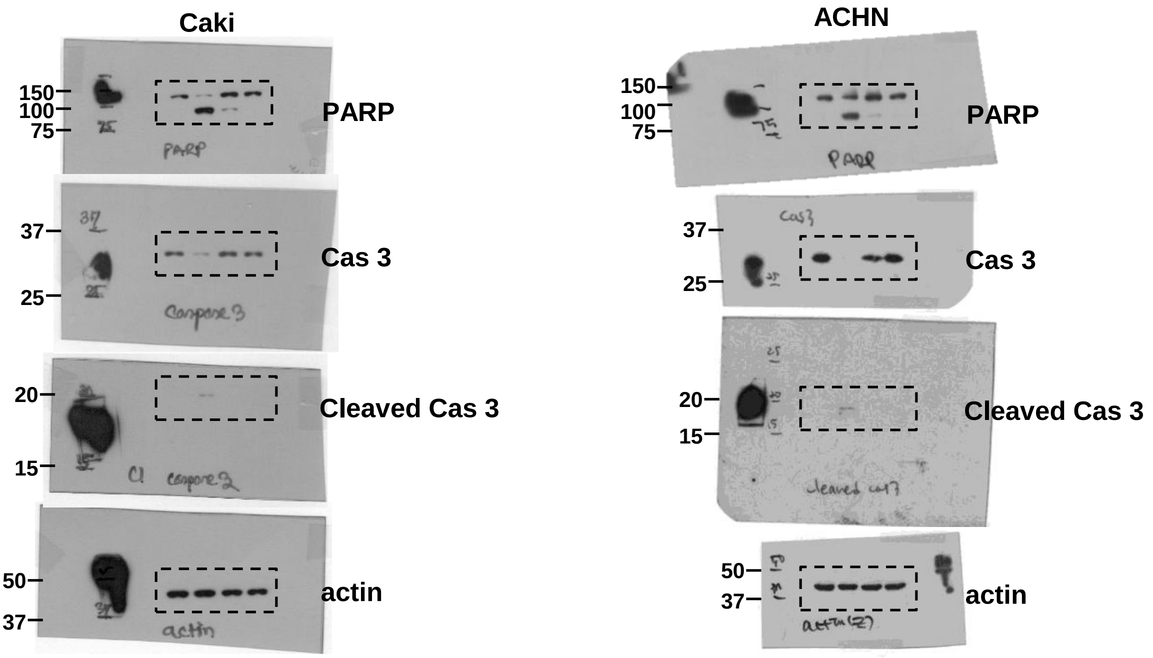

Figure S2. Uncropped western blots for Figure 1.

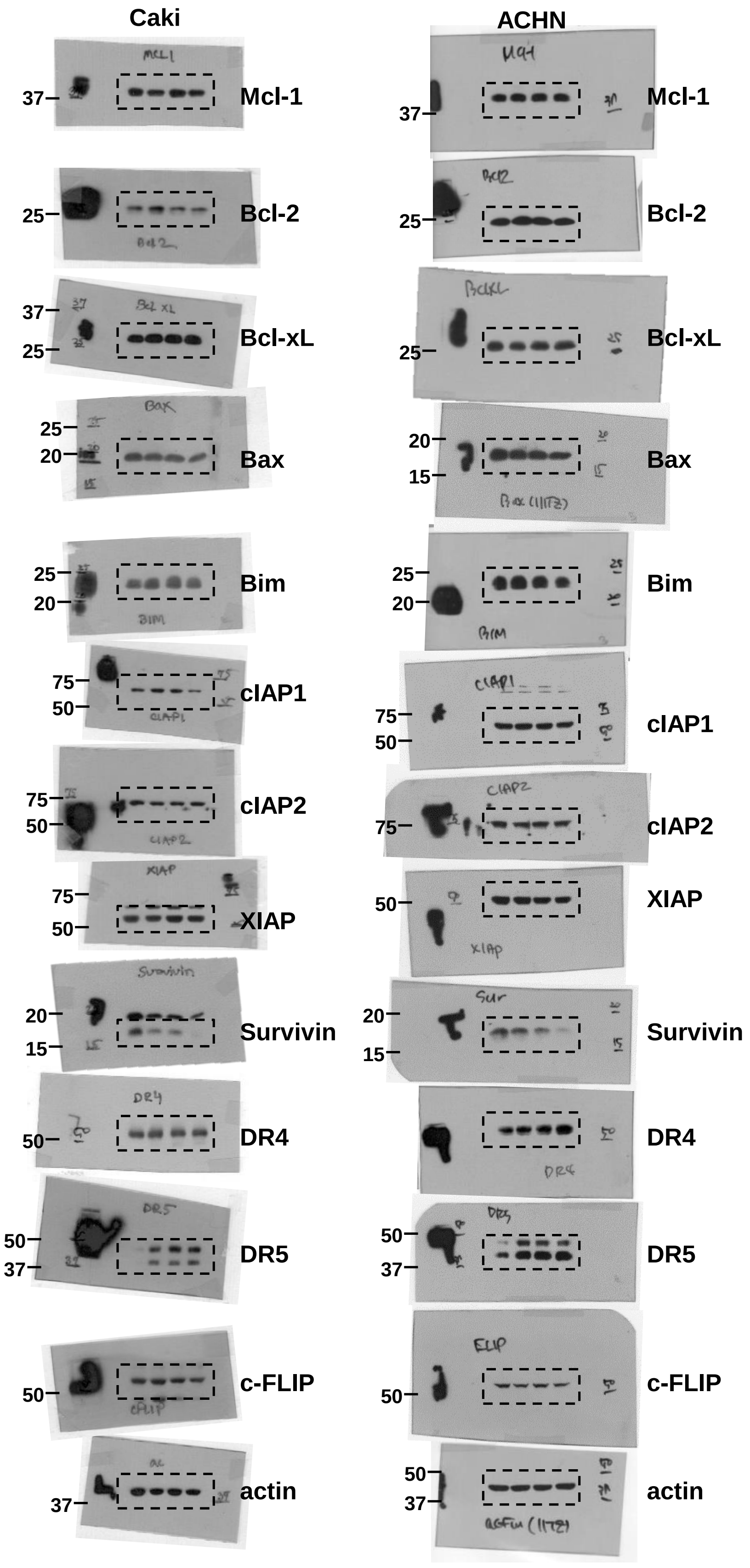

Figure S3. Uncropped western blots for Figure 2.

Figure 3a

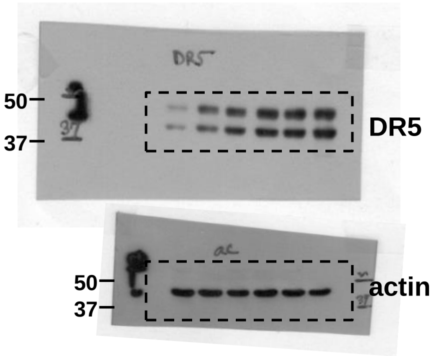

Figure 3d

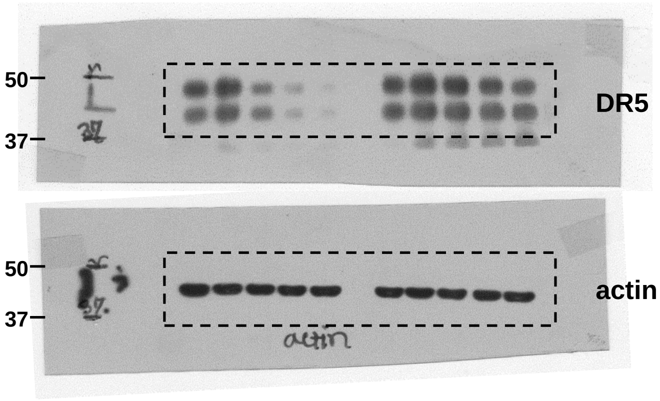

Figure 3e

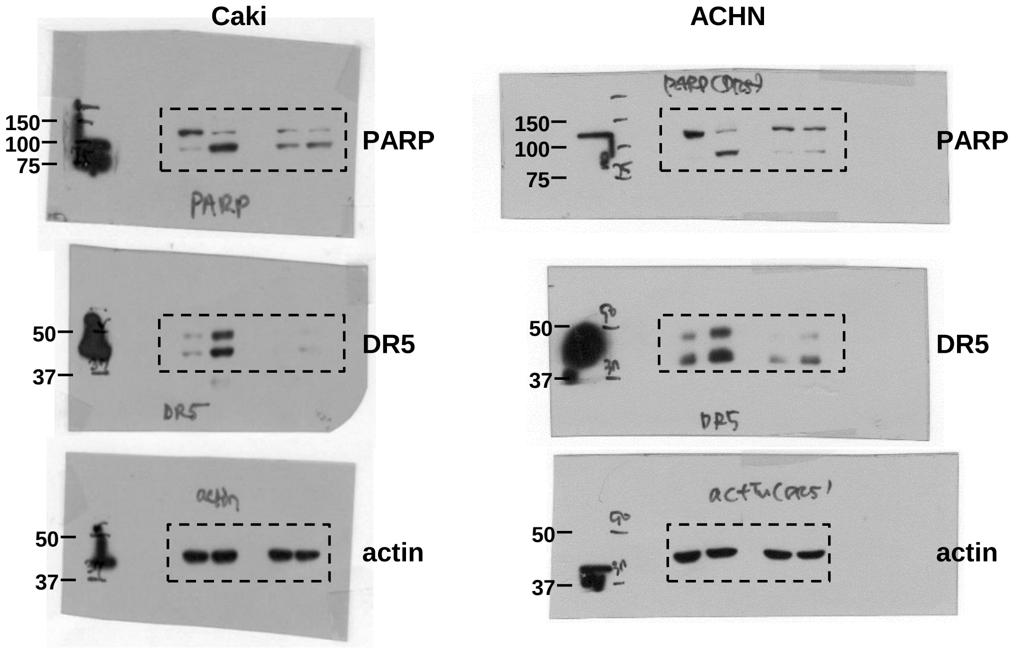

Figure S4. Uncropped western blots for Figure 3.

Figure 4a

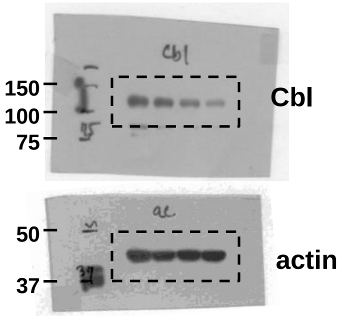

Figure 4b

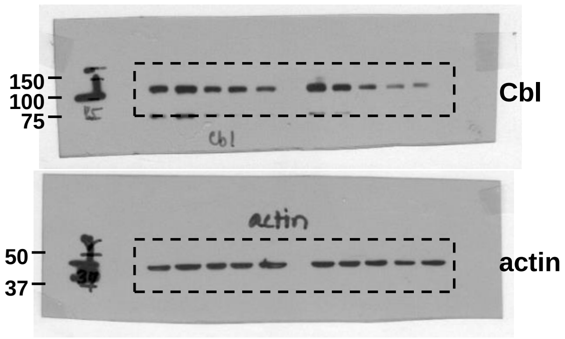

Figure 4c

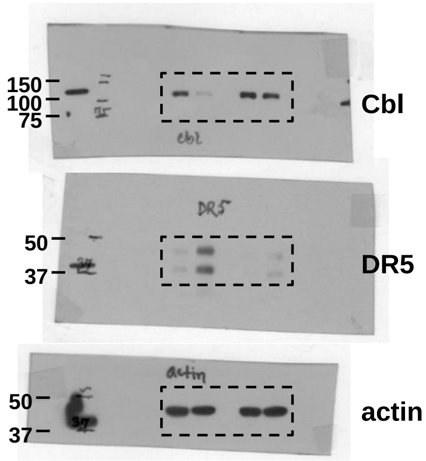

Figure 4d

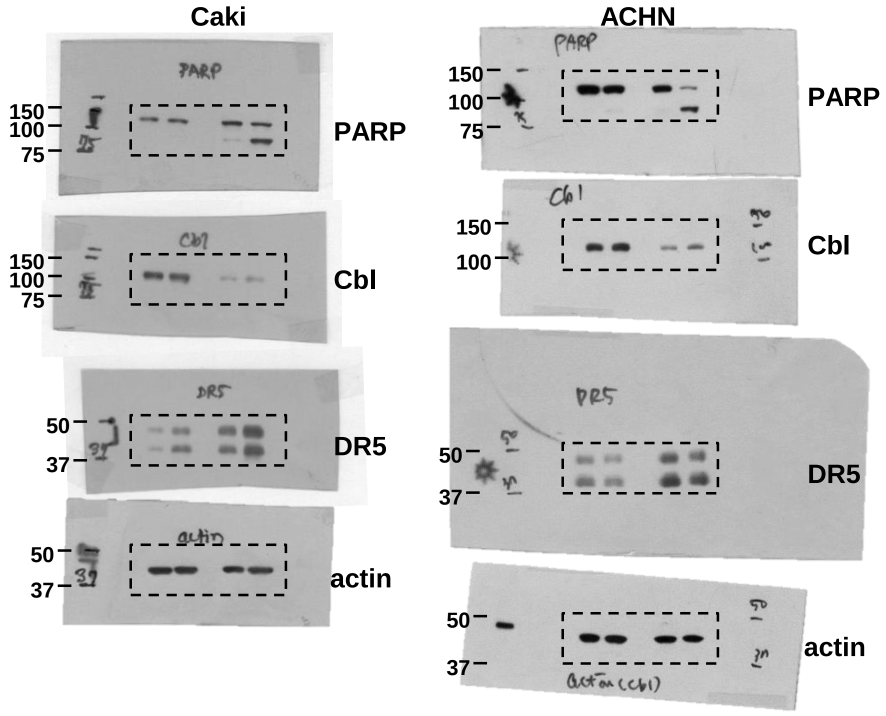

Figure S5. Uncropped western blots for Figure 4.

Figure 5a

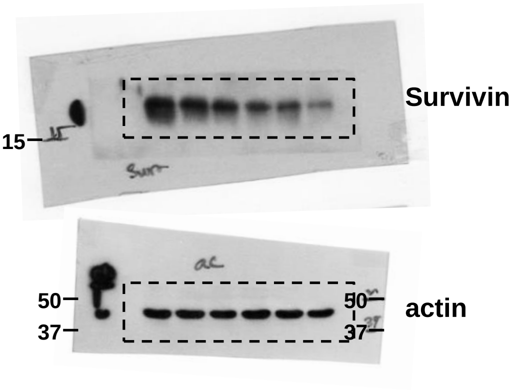

Figure 5b

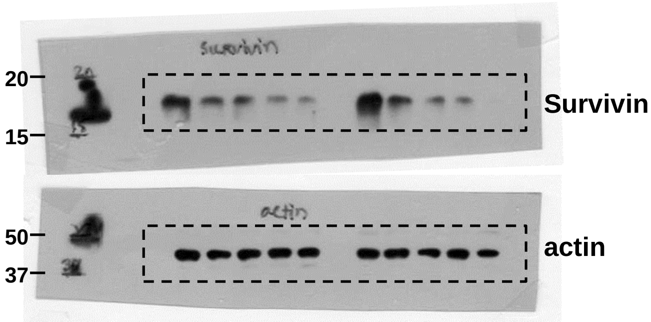

Figure 5c

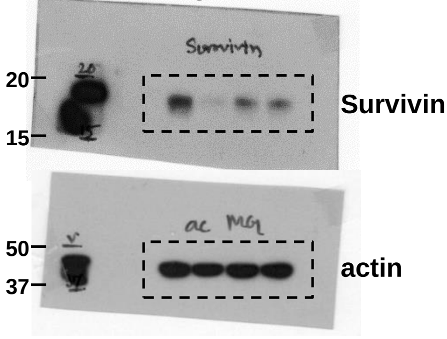

Figure 5d

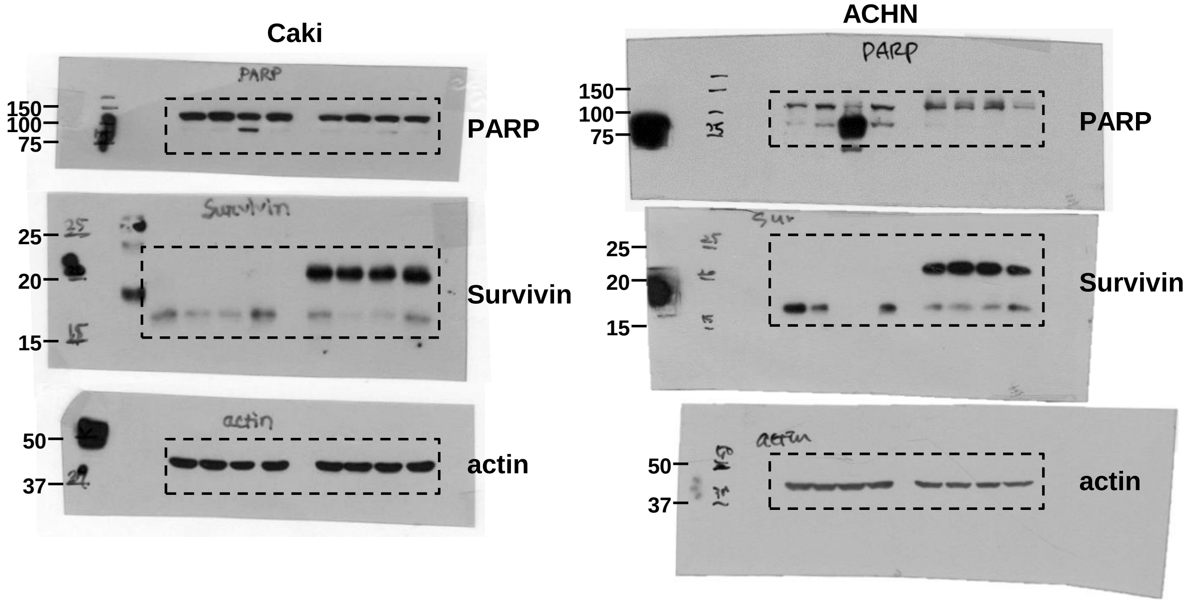

Figure S6. Uncropped western blots for Figure 5.

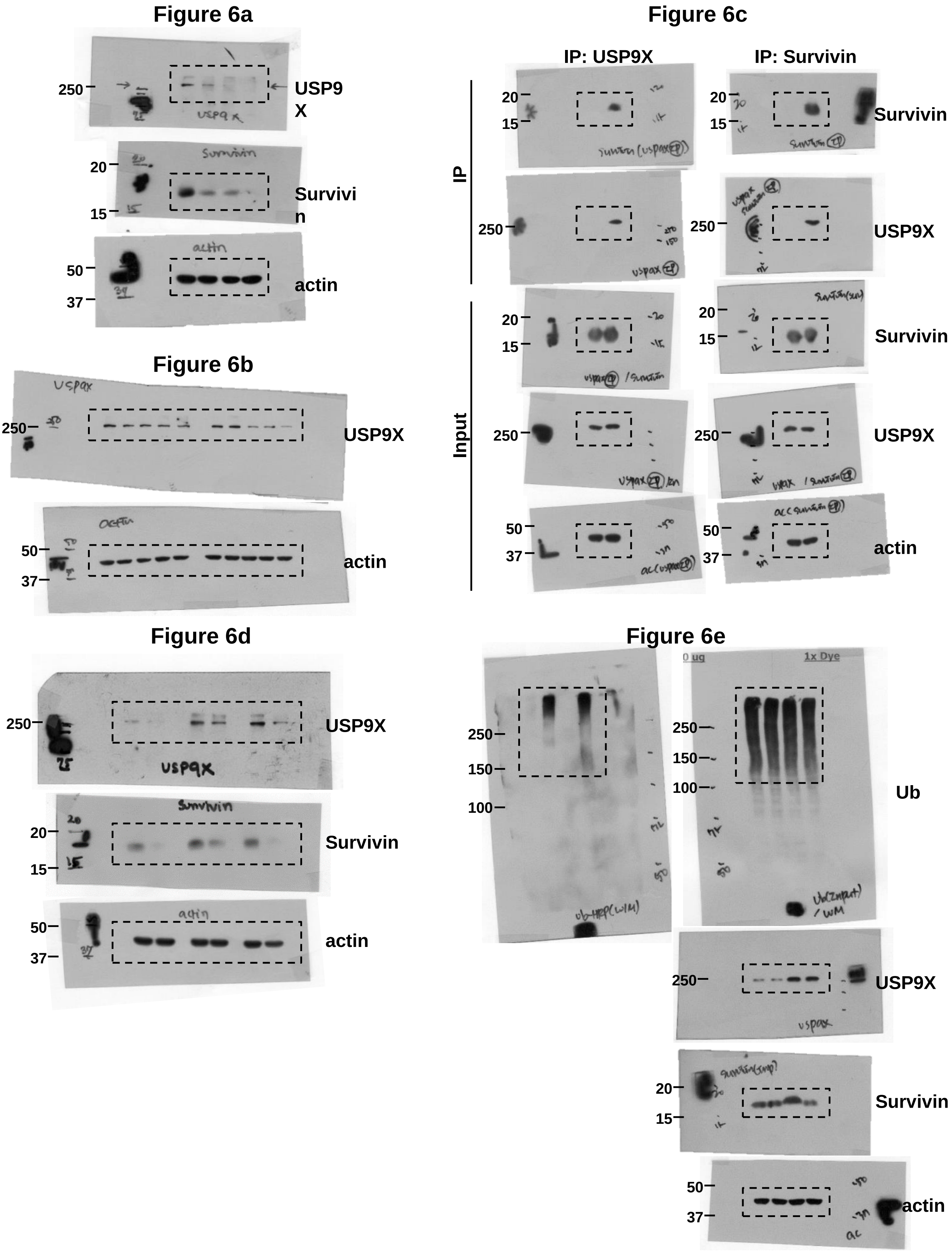

Figure S7. Uncropped western blots for Figure 6.

Figure 7a

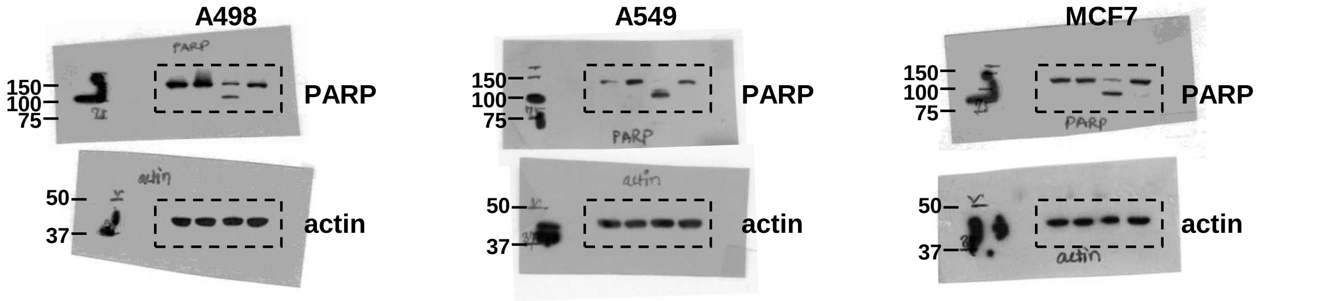

Figure 7b

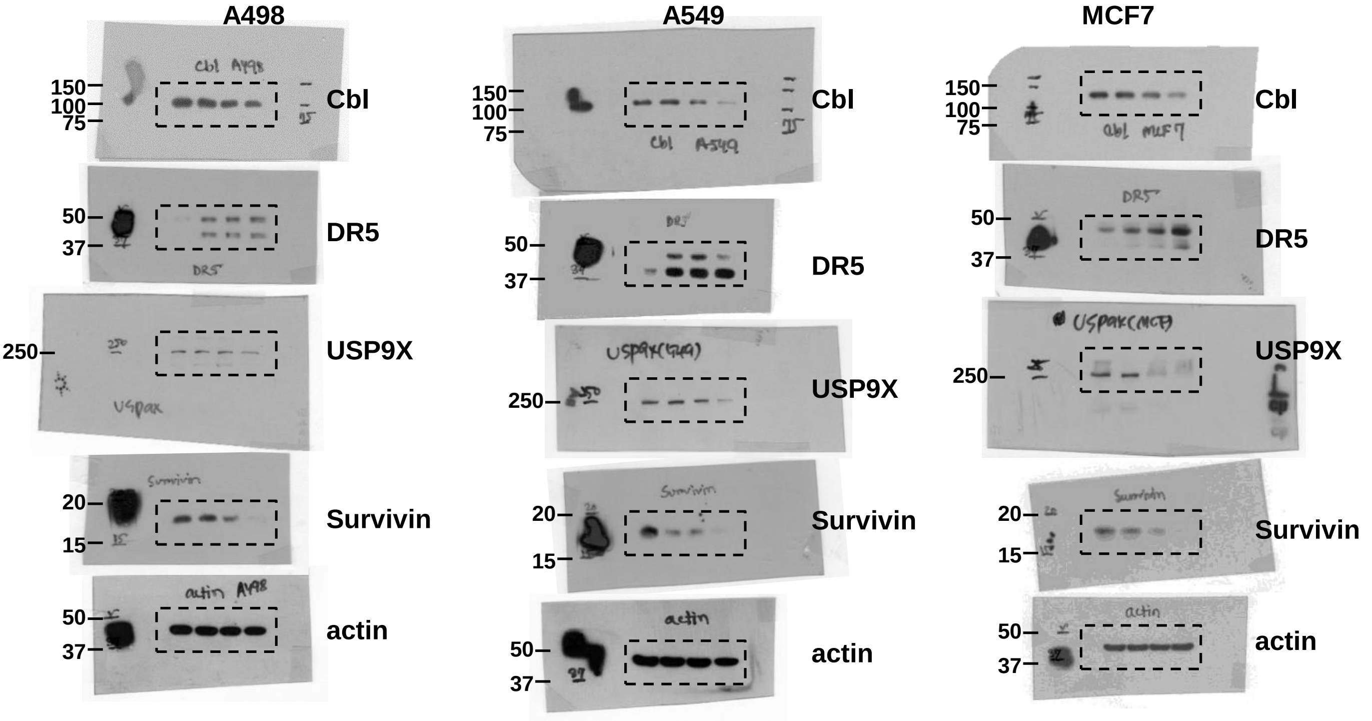

Figure 7d

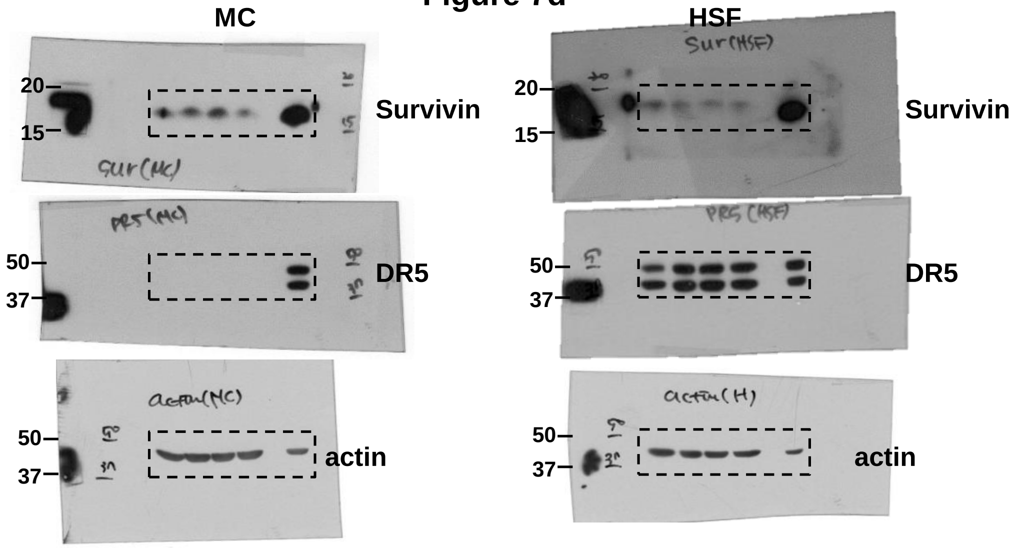

Figure S8. Uncropped western blots for Figure 7.

Methods

University of California, Santa Cruz (UCSC) Xena

The mRNA data for kidney clear carcinoma tissues and normal kidney tissues were downloaded from the UCSC Xena public data hub (https://xena.ucsc.edu) [1,2].The mRNA expression of survivin (BIRC5), DR5 (TNFRSF10B), USP9X and Cbl were obtained from the TCGA Kidney Clear Cell Carcinoma (KIRC) cohort and analyzed using UCSC database. The overall survival of survivin (BIRC5), DR5 (TNFRSF10B), USP9X and Cbl was generated by the UCSC database for Kaplan-Meier analysis of 534 patients with high versus low expression.

References

1. Goldman, M. J.; Craft, B.; Hastie, M.; Repecka, K.; McDade, F.; Kamath, A.; Banerjee, A.; Luo, Y.; Rogers, D.; Brooks, A. N.; Zhu, J.; Haussler, D. Visualizing and interpreting cancer genomics data via the Xena platform. Nat. Biotechnol. 2020, 38, 675-678.

2. Goldman, M.; Craft, B.; Kamath, A.; Brooks, A.; Zhu, J.; Haussler, D. The UCSC Xena Platform for cancer genomics data visualization and interpretation. BioRxiv. 2018.
